# Distinct involvement of the cranial and spinal nerves in progressive supranuclear palsy

**DOI:** 10.1093/brain/awad381

**Published:** 2023-11-16

**Authors:** Hidetomo Tanaka, Ivan Martinez-Valbuena, Shelley L Forrest, Blas Couto, Nikolai Gil Reyes, Alonso Morales-Rivero, Seojin Lee, Jun Li, Ali M Karakani, David F Tang-Wai, Charles Tator, Mozhgan Khadadadi, Nusrat Sadia, Maria Carmela Tartaglia, Anthony E Lang, Gabor G Kovacs

**Affiliations:** Department of Laboratory Medicine and Pathobiology and Tanz Centre for Research in Neurodegenerative Disease, University of Toronto, Toronto, Ontario M5T 0S8, Canada; Department of Laboratory Medicine and Pathobiology and Tanz Centre for Research in Neurodegenerative Disease, University of Toronto, Toronto, Ontario M5T 0S8, Canada; Department of Laboratory Medicine and Pathobiology and Tanz Centre for Research in Neurodegenerative Disease, University of Toronto, Toronto, Ontario M5T 0S8, Canada; Laboratory Medicine Program and Krembil Brain Institute, University Health Network, Toronto, Ontario M5T 0S8, Canada; Edmond J. Safra Program in Parkinson's Disease, Rossy Program for PSP Research and the Morton and Gloria Shulman Movement Disorders Clinic, Toronto Western Hospital, Toronto, Ontario M5T 2S8, Canada; Edmond J. Safra Program in Parkinson's Disease, Rossy Program for PSP Research and the Morton and Gloria Shulman Movement Disorders Clinic, Toronto Western Hospital, Toronto, Ontario M5T 2S8, Canada; University Health Network Memory Clinic, Toronto Western Hospital, Toronto, Ontario M5T 2S8, Canada; Department of Laboratory Medicine and Pathobiology and Tanz Centre for Research in Neurodegenerative Disease, University of Toronto, Toronto, Ontario M5T 0S8, Canada; Department of Laboratory Medicine and Pathobiology and Tanz Centre for Research in Neurodegenerative Disease, University of Toronto, Toronto, Ontario M5T 0S8, Canada; Department of Laboratory Medicine and Pathobiology and Tanz Centre for Research in Neurodegenerative Disease, University of Toronto, Toronto, Ontario M5T 0S8, Canada; University Health Network Memory Clinic, Toronto Western Hospital, Toronto, Ontario M5T 2S8, Canada; Department of Medicine/Division of Neurology, University of Toronto, Toronto, Ontario M5S 3H2, Canada; Krembil Brain Institute, Toronto Western Hospital, Toronto, Ontario M5T 2S8, Canada; Krembil Brain Institute, Toronto Western Hospital, Toronto, Ontario M5T 2S8, Canada; Canadian Concussion Centre, Krembil Brain Institute, University Health Network, Toronto, Ontario M5T 0S8, Canada; Canadian Concussion Centre, Krembil Brain Institute, University Health Network, Toronto, Ontario M5T 0S8, Canada; Canadian Concussion Centre, Krembil Brain Institute, University Health Network, Toronto, Ontario M5T 0S8, Canada; University Health Network Memory Clinic, Toronto Western Hospital, Toronto, Ontario M5T 2S8, Canada; Department of Medicine/Division of Neurology, University of Toronto, Toronto, Ontario M5S 3H2, Canada; Krembil Brain Institute, Toronto Western Hospital, Toronto, Ontario M5T 2S8, Canada; Canadian Concussion Centre, Krembil Brain Institute, University Health Network, Toronto, Ontario M5T 0S8, Canada; Tanz Centre for Research in Neurodegenerative Diseases, University of Toronto, Toronto, Ontario M5T 0S8, Canada; Edmond J. Safra Program in Parkinson's Disease, Rossy Program for PSP Research and the Morton and Gloria Shulman Movement Disorders Clinic, Toronto Western Hospital, Toronto, Ontario M5T 2S8, Canada; Department of Medicine/Division of Neurology, University of Toronto, Toronto, Ontario M5S 3H2, Canada; Krembil Brain Institute, Toronto Western Hospital, Toronto, Ontario M5T 2S8, Canada; Department of Laboratory Medicine and Pathobiology and Tanz Centre for Research in Neurodegenerative Disease, University of Toronto, Toronto, Ontario M5T 0S8, Canada; Department of Medicine/Division of Neurology, University of Toronto, Toronto, Ontario M5S 3H2, Canada; Krembil Brain Institute, Toronto Western Hospital, Toronto, Ontario M5T 2S8, Canada; Laboratory Medicine Program, University Health Network, Toronto, Ontario M5T 0S8, Canada

**Keywords:** cranial nerve, peripheral nervous system, progressive supranuclear palsy, spinal nerve, tauopathy

## Abstract

The most frequent neurodegenerative proteinopathies include diseases with deposition of misfolded tau or α-synuclein in the brain. Pathological protein aggregates in the PNS are well-recognized in α-synucleinopathies and have recently attracted attention as a diagnostic biomarker. However, there is a paucity of observations in tauopathies. To characterize the involvement of the PNS in tauopathies, we investigated tau pathology in cranial and spinal nerves (PNS-tau) in 54 tauopathy cases [progressive supranuclear palsy (PSP), *n* = 15; Alzheimer’s disease (AD), *n* = 18; chronic traumatic encephalopathy (CTE), *n* = 5; and corticobasal degeneration (CBD), *n* = 6; Pick’s disease, *n* = 9; limbic-predominant neuronal inclusion body 4-repeat tauopathy (LNT), *n* = 1] using immunohistochemistry, Gallyas silver staining, biochemistry, and seeding assays. Most PSP cases revealed phosphorylated and 4-repeat tau immunoreactive tau deposits in the PNS as follows: (number of tau-positive cases/available cases) cranial nerves III: 7/8 (88%); IX/X: 10/11 (91%); and XII: 6/6 (100%); anterior spinal roots: 10/10 (100%). The tau-positive inclusions in PSP often showed structures with fibrillary (neurofibrillary tangle-like) morphology in the axon that were also recognized with Gallyas silver staining. CBD cases rarely showed fine granular non-argyrophilic tau deposits. In contrast, tau pathology in the PNS was not evident in AD, CTE and Pick’s disease cases. The single LNT case also showed tau pathology in the PNS. In PSP, the severity of PNS-tau involvement correlated with that of the corresponding nuclei, although, occasionally, p-tau deposits were present in the cranial nerves but not in the related brainstem nuclei. Not surprisingly, most of the PSP cases presented with eye movement disorder and bulbar symptoms, and some cases also showed lower-motor neuron signs. Using tau biosensor cells, for the first time we demonstrated seeding capacity of tau in the PNS. In conclusion, prominent PNS-tau distinguishes PSP from other tauopathies. The morphological differences of PNS-tau between PSP and CBD suggest that the tau pathology in PNS could reflect that in the central nervous system. The high frequency and early presence of tau lesions in PSP suggest that PNS-tau may have clinical and biomarker relevance.

## Teaser

Tanaka *et al.* investigate whether tau pathology is present in the cranial and spinal nerves of individuals with different tauopathies, and find that prominent peripheral nervous system tau distinguishes progressive supranuclear palsy from other tau-based disorders.

## Introduction

Neurodegenerative diseases are characterized by the progressive dysfunction and loss of neurons associated with the deposition of misfolded proteins mostly in the CNS.^1^ The most frequent forms include those with misfolded tau or α-synuclein. Progressive supranuclear palsy (PSP) and corticobasal degeneration (CBD) are the main forms of idiopathic tauopathies. Additional forms include Alzheimer’s disease (AD), which is an extracellular filamentous protein (amyloid-β, Aβ) associated tauopathy and chronic traumatic encephalopathy (CTE).^2^ These conditions are distinguished by the predominant tau isoform [four or three repeat (R) or combined] and distinct structure of tau filaments.^3^ The involvement of the PNS in neurodegenerative proteinopathies is well-recognized in α-synucleinopathies. Parkinson’s disease/Lewy body disease (PD/LBD) often shows non-motor symptoms, including sensory and autonomic disturbances, and the aggregation of α-synuclein in the PNS is frequently documented.^4,5^ Moreover, recent studies have suggested that using a novel and ultrasensitive seeding assay, the real-time quaking-induced conversion (RT-QuIC), seeding activity of α-synuclein can be measured in the skin, which may present a novel non-invasive diagnostic assay for PD and other synucleinopathies.^6,7^ PNS involvement is increasingly important due to its *in vivo* biomarker relevance. Recent studies have begun to capture involvement of the PNS in tauopathies such as autonomic and sensory disturbances, electrophysiological features, and pathological features, namely loss of cutaneous nerve fibres.^8-10^ However, only a few studies have reported the expression of phosphorylated tau (p-tau) immunoreactivity in peripheral nerves.^5,11-13^ In the present study, we investigated the involvement of PNS tau pathology in tauopathies in a wide range of cranial and spinal nerves and found clear evidence of 4R and p-tau immunoreactive deposits associated with seeding capacity in peripheral nerves, particularly in PSP.

## Materials and methods

### Subjects

Fifty-four autopsy cases were selected in this study, including 15 cases of PSP, 18 cases of AD, five cases of CTE, six cases of CBD, nine cases of Pick’s disease and one case of limbic-predominant neuronal inclusion body 4-repeat tauopathy (LNT)^14^ from the University Health Network–Neurodegenerative Brain Collection (UHN–NBC, Toronto, Canada) that came to autopsy between 1982 and 2022, based on a definite neuropathological diagnosis. AD cases were included if there was severe AD neuropathologic change,^15^ and it constituted the primary CNS pathology. One case exhibited severe AD pathology and mild CTE pathology^16^ but was categorized in the AD group due to the predominant AD pathology (Table 1 and Supplementary Table 1). Autopsy tissue from human brains was collected with the informed consent of patients or their relatives and the approval of the local institutional review board. This study was approved by the University Health Network Research Ethics Board (Nr. 20–5258). Prior to inclusion in the study, a systematic neuropathological examination was performed following established diagnostic criteria of neurodegenerative conditions and co-pathologies.^15,17-20^

**Table 1:** Summary of tau pathology in the PNS in tauopathies

	PSP (*n* = 15)			AD (*n* = 18)			CTE (*n* = 5)			CBD (*n* = 6)		
Number of cases	Avail	Tau	%	Avail	Tau	%	Avail	Tau	%	Avail	Tau	%
**Cranial nerves**												
III	8	7	88	10	0	0***	2	0	0	4	1	25
V	1	1	100	na	na	na	1	0	0	2	0	0
IX/X	11	10	91	5	0	0**	2	0	0*	5	1	20*
XI	4	4	100	na	na	na	na	na	na	1	0	0
XII	6	6	100	4	0	0**	2	0	0*	1	0	0
**Spinal roots**												
Ant.	10	10	100	5	0	0***	3	0	0**	3	1	33*
Post.	5	3	60	5	0	0	3	0	0	2	0	0
**Total case number**	15	15	100	14	0	0***	5	0	0***	5	1	20**

AD = Alzheimer’s disease; Ant. = anterior spinal roots; Avail = number of available cases; CBD = corticobasal degeneration; CTE = chronic traumatic encephalopathy; na = not available; Post. = posterior spinal roots; PSP = progressive supranuclear palsy; Tau = number of tau-positive cases. *P < 0.05; **P < 0.01; ***P < 0.0001 (versus PSP cases, Fisher’s exact test).

### Neuropathological examination

Histopathological analysis was carried out using formalin-fixed, paraffin-embedded, 4.5-μm thick sections from the midbrain, pons, medulla oblongata, and spinal cord (cervical, thoracic, lumbar segments, and cauda equina). Oculomotor and trigeminal nerves were examined at the level of the midbrain and pons, respectively. Glossopharyngeal and vagus nerves were examined at the level of the dorsal vagal nucleus. Since it was difficult to differentiate the glossopharyngeal nerve from the vagus nerve on the sections, both nerves were described as a unit.^21^ Accessory nerves were examined at the level of the supraspinal nucleus (spinal accessory nucleus) and the pyramidal decussation. Hypoglossal nerves were examined at the level of the hypoglossal nucleus.

Histological examination was performed using two stains: Luxol fast blue/haematoxylin and eosin (LFB/H&E) as well as Gallyas–Braak (G-B). Immunohistochemistry was performed using the following primary antibodies: p-tau (clone AT8, pSer202/Thr205, 1:1000, Invitrogen/ThermoFisher; clone AT180, pThr231, 1:1000, Invitrogen/ThermoFisher; Phospho-Tau Thr217, 1:1000, Invitrogen/ThermoFisher); misfolded tau (Alz-50, 1:100, Dr Peter Davies); Aβ (Clone 6F/3D, 1:50, Dako/Agilent); α-synuclein (clone 5G4, 1:4000, Analytikjena)^22^; phosphorylated TDP-43 (clone 11-9, 1:2000, CosmoBio); and p62 (clone 3/P62 LCK LIGAND, 1:500, BD Transduction Laboratories). Immunostaining for 3-R-tau (RD3, clone 8B6/C11, 1:2000, MilliporeSigma) and 4-R-tau (RD4, clone 1E1/A6, 1:200, MilliporeSigma) was additionally performed in cases that exhibited immunoreactivity for AT8 in the PNS. Antigen retrieval was performed using Dako PT Link with low pH solution (anti-p-tau, α-synuclein, TDP-43, p62, 3R- and 4R-tau antibodies) and/or 80% formic acid (1 h for anti-Aβ, 5 min for anti-α-synuclein and 1 min for anti-TDP-43, 3R- and 4R-tau antibodies). Immunostaining was performed using the Dako Autostainer Link 48 and EnVision FLEX+ Visualization System, according to manufacturer’s instructions. Subsequently, all sections were counterstained with haematoxylin.

Semiquantitative analysis of the severity of tau-related pathology in the peripheral nerves was assessed in each region (70 000 μm^2^) with the ‘PMA.start’ software, whole slide image viewer for digital pathology (Pathomation, Belgium), using AT8-immunostained sections, which were scanned at a magnification of ×40 with a TissueScope LE120 slide scanner (Huron Digital Pathology). The numbers of tau-positive fibres were estimated as: −, none; +, 2 to 9; ++, 10 to 29; +++, 30 to 49; and ++++, over 50. The severity of tau-related pathology in the brainstem nuclei and spinal grey matter was also assessed as: −, absent; +, minimal; ++, mild; +++, moderate; and ++++, severe.

A double-labelling immunofluorescence study was performed on sections obtained from the medulla oblongata and spinal cord with the peripheral nerves (IX/X nerves and anterior spinal roots) in two representative PSP cases using a mixture of a rabbit polyclonal antibody against phosphorylated tau (pThr217, 1:500) and mouse monoclonal antibody against phosphorylated neurofilament (p-NF, clone SMI31, BioLegend, 1:2000) or neurofilament (NF, clone 2F11, Dako/Agilent,1:2000) or a mixture of rabbit monoclonal antibody against S100β (ab52642, Abcam, 1:10 000) and mouse monoclonal antibody against p-tau (AT8, 1:1000). The secondary antibodies used were Alexa Fluor 488 donkey anti-mouse IgG (Invitrogen/ThermoFisher, 1:500) and Alexa Fluor 555 goat anti-rabbit IgG (Invitrogen/ThermoFisher, 1:500). The sections were treated with Sudan black B and sodium borohydride to suppress autofluorescence. Nuclei were counterstained with spectral 4′,6-diamidino-2-phenylindole (DAPI; Akoya Bioscience). Double-labelled immunofluorescence imaging was performed using a Nikon C2Si+ confocal on a Nikon Ti2-E inverted microscope. Images were captured using NIS-Elements (v5.30.04). In this double-labelling immunofluorescence study, the peripheral nerves were evaluated as far away (∼>5 mm) from the brainstem and spinal cord as possible, to examine ‘peripheral myelin’.^23-26^

### Protein extraction and western blot analysis

Using a 4-mm tissue punch, microdissection of the motor cortex and the spinal roots (at the cervical cord level) was performed in two PSP cases (PSP Case 8 and PSP Case 10) as previously described.^27^ All the punches were stored in low protein binding tubes (Eppendorf) and immediately flash-frozen and stored at −80°C. Frozen microdissected tissue (30–50 mg) was thawed on wet ice and then immediately homogenized in 500 μl of PBS spiked with protease (Roche) and phosphatase inhibitors (Thermo Scientific) in a gentle-MACS Octo Dissociator (Miltenyi BioTec). The homogenate was transferred to a 1.5-ml low protein binding tube (Eppendorf) and centrifuged at 10 000*g* for 10 min at 4°C as previously described.^27^ The supernatant was collected and aliquoted in 0.5-ml low protein binding tubes (Eppendorf) to avoid excessive freeze–thaw cycles. A bicinchoninic acid protein (BCA) assay (Thermo Scientific) was performed to determine the total protein concentration of all samples 30 μg of total protein was used for western blotting. Gel electrophoresis was performed using a NuPAGE 4–12% Bis-Tris gel (Thermo Scientific). Proteins were transferred to 0.45-μm nitrocellulose membranes for 60 min at 35 V. The membrane was blocked for 60 min at room temperature in blocking buffer [5% w/v skimmed milk in 1× Tris-buffered saline and 0.05% v/v Tween-20 (TBST)] and then incubated overnight at 4°C with a primary antibody directed against total tau (HT7 clone, mid-domain of tau at amino acid 159–163 1:1000 dilution, ref: MN1000, ThermoFisher) diluted in the blocking buffer. The membrane was washed three times with TBST and then incubated for 60 min at room temperature with horseradish peroxidase-conjugated secondary antibody (1:3000 dilution, ref: 172-1011, Bio-Rad) in the blocking buffer. Following another three washes with TBST, immunoblots were developed using enhanced chemiluminescence (ECL) western blotting detection reagents (Amersham) and imaged using X-ray films.

### *In vitro* tau seeding fluorescence resonance energy transfer-biosensor assay

The *in vitro* seeding assay was performed as previously described.^28,29^ Briefly, the Tau RD P301S fluorescence resonance energy transfer (FRET) biosensor (ATCC CRL-3275) cells were cultured at 37°C in 5% CO_2_ in Dulbecco’s modified Eagle medium (DMEM), 10% v/v fetal bovine serum and 0.5% v/v penicillin-streptomycin. Cells were plated on Ibidi clear-bottom 96-well plates at a density of 40 000 cells per well. Human tissue homogenates (12 µg of total protein per well), from the motor cortex and the spinal roots in two PSP cases (PSP Cases 8 and 10) were then incubated with Lipofectamine 2000 (Invitrogen; final concentration 1% v/v) in Opti-MEM for 10 min at room temperature before being added to the cells. Each brain region was tested in duplicates. After 48 h, cells were fixed with 4% paraformaldehyde solution in PBS (Thermo Scientific) for 15 min and stained 10 min with NucBlue^TM^ Fixed Cell ReadyProbes^TM^ Reagent (DAPI; Invitrogen) at room temperature. The cells were imaged with a 20× objective using a Nikon ECLIPSE Ti2 confocal microscope.

### Statistical analysis

The clinical and pathological findings were analysed using SPSS 26.0 (IBM, Armonk, NY, USA), with the significance level set at 0.05. Fisher’s exact test was used for qualitative variables. Quantitative variables (age at death and duration of illness) were compared using the Kruskal–Wallis test with *post hoc* Bonferroni correction.

## Results

### Comparison of tau pathology in the PNS among tauopathies

The presence of tau pathology, characterized by p-tau deposits, was evaluated in the extramedullary segment of cranial nerves (III, V, IX/X, XI and XII) and spinal nerve roots (anterior and posterior roots) (Table 1).

**Figure 1:**
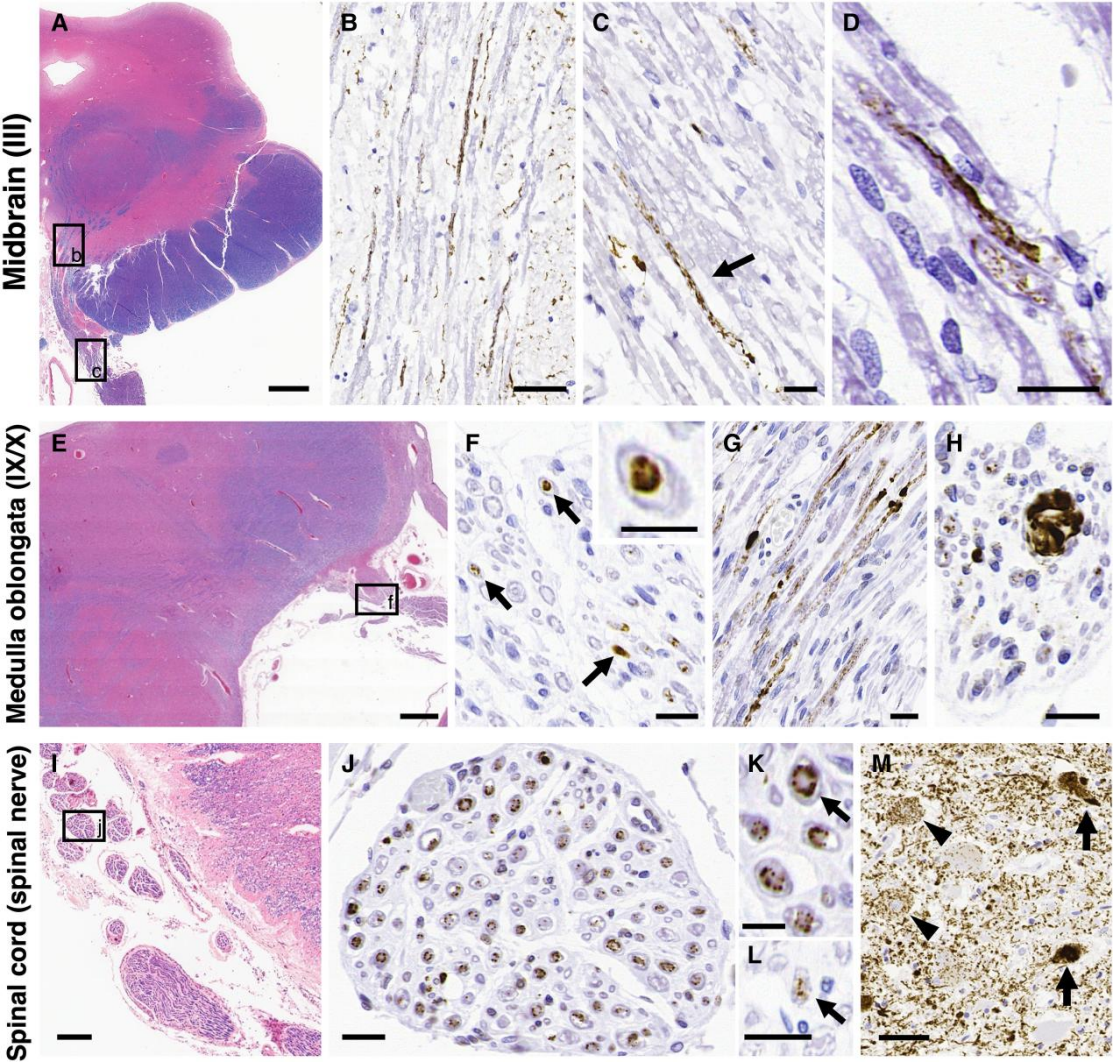
**Representative photomicrographs of tau pathology in the PNS in progressive supranuclear palsy**. (**A**–**D**) In the midbrain (**A**), phosphorylated tau deposits are evident in the oculomotor nerves: III, intra-medullary fibres (**B**: high magnification image of black box ‘b’ in **A**) and extra-medullary fibres [**C**: high magnification image of black box ‘c’ in **A**; arrow: phosphorylated-tau (p-tau) deposits; and **D**]. (**E**–**H**) In the medulla oblongata (**E**), tau accumulates in the glossopharyngeal/vagus nerves: IX/X, cross sections (**F**: high magnification image of black box ‘f’ in **E**; arrows: p-tau deposits; enlarged image in the *top right* corner) and sagittal sections (**G**). Occasionally, large inclusions with fibrillary morphology are also seen (**H**). (**I**–**M**) There are massive amounts of p-tau deposits in the spinal anterior roots [progressive supranuclear palsy (PSP) Case 1; **J**: high magnification image of black box ‘j’ in **I**; and **K**: high magnification image of ‘J’, arrow: p-tau deposits primarily located externally) and small amounts of p-tau deposits in the posterior roots (**L**, arrow; PSP Case 3) at the cervical cord level. (**M**) Tau pathology is also evident in the anterior horn of the cervical cord, PSP Case 1 (arrow: neurofibrillary tangle-like inclusions; and arrowhead: pre-tangle-like inclusions). (**A**–**C**) PSP Case 15, (**D**, **G–K** and **M**) PSP Case 1, (**E** and **F**) PSP Case 11 and (**L**) PSP Case 3. (**A, E** and **I**) Luxol fast blue/haematoxylin and eosin (LFB/H&E) and (**B**–**D**, **F**–**H** and **J**–**M**) AT8. Scale bars in **A** = 2 mm, **B** and **M** = 50 μm, **C**, **D**, **F**–**H**, **J** and **L** = 20 μm, **E** = 1 mm, **I** = 200 μm and **K** and enlarged image of the *top right* corner in **F** = 10 μm.

PSP cases differed from other forms of tauopathies in terms of a significantly higher frequency (around 90%–100%) of tau pathology in the PNS (Table 1). Specifically, the number of p-tau-positive cases/available cases in cranial nerves III was 7/8 (88%) (Fig. 1A–D), IX/X was 10/11 (91%) (Fig. 1E–H), XI was 4/4 (100%), XII was 6/6 (100%) and anterior spinal roots was 10/10 (100%) (Fig. 1I–K). Tau pathology was observed in the IX/X nerves and V nerves (1/1, 100%) composed of both motor and sensory nerves, as well as in posterior spinal roots (3/5, 60%), which purely consisted of sensory nerves (Fig. 1L). In contrast to PSP cases, no tau pathology was evident in the PNS of AD or CTE cases: AD cases cranial nerves III (0/10: 0%), IX/X (0/5: 0%), XII (0/4: 0%), anterior spinal roots (0/5: 0%) and posterior spinal roots (0/5: 0%); CTE cases cranial nerves III (0/2: 0%), V (0/1: 0%), IX/X (0/2: 0%), XII (0/2: 0%), anterior spinal roots (0/3: 0%) and posterior spinal roots (0/3: 0%). CBD cases only exhibited a few tau deposits, namely in cranial nerves III (1/4: 25%), IX/X (1/5: 20%) and anterior spinal roots (1/3: 33%) (Table 1). In Pick’s disease cases, there were no tau deposits in the PNS (Supplementary Table 2). The single 4-repeat tauopathy LNT case exhibited PNS-tau lesions in the anterior spinal roots at the three levels, namely cervical, thoracic, and lumbar cord levels (Supplementary Fig. 1).

### Morphological features of PNS-tau pathology in progressive supranuclear palsy

**Figure 2:**
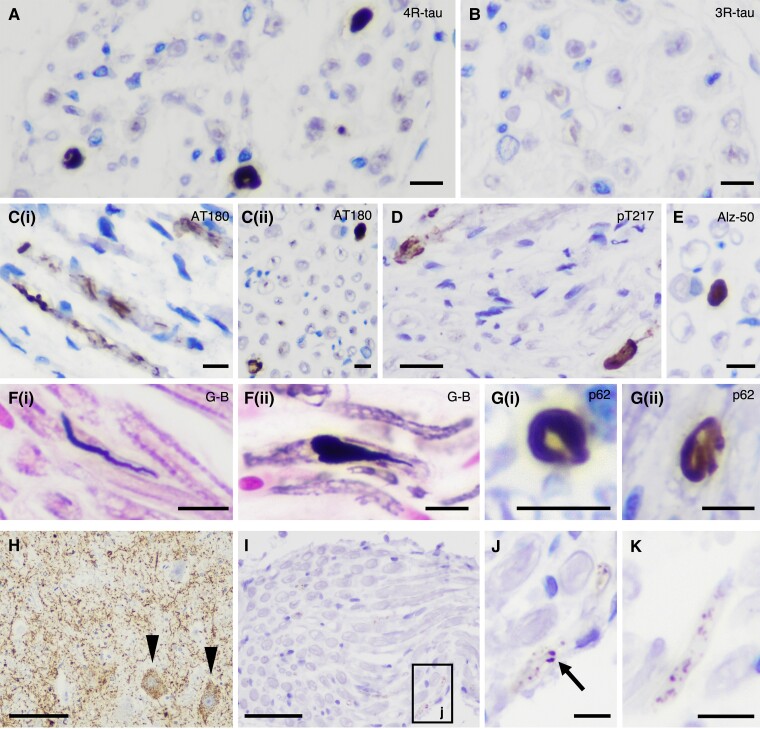
**Staining profiles of PNS-tau in progressive supranuclear palsy (PSP) and comparing morphological features of tau-positive inclusions in PSP and corticobasal degeneration (CBD)**. (**A**–**G**) Representative photomicrographs of staining profiles of the PNS-tau in PSP. The tau-positive inclusions in the PNS are detected with 4-repeat tau specific antibody (RD4) (**A**) but not with 3-repeat tau-specific antibody (RD3) (**B**). The tau-positive inclusions are also recognized with several phosphorylated tau antibodies [**C**(**i** and **ii**): AT180 and **D**: pThr217] other than AT8 and also Alz-50, which is for misfolded tau (**E**). The inclusions in PSP often show large structures with fibrillary morphology (**A** and **E**), which are stained with Gallyas–Braak silver staining (G-B) [**F**(**i** and **ii**)]. Some inclusions also show p62-immunoreactivity [**G**(**i** and **ii**)]. (**H**–**K**) Representative photomicrographs of tau pathology in the PNS and related nuclei in CBD Case 3, namely anterior horn (**H**), spinal anterior roots (**I** and **J**: high magnification image of black box ‘j’ in **I**) in the cervical cord and IX/X nerve (**K**). Although this CBD case shows massive tau pathology in the anterior horn of the cervical cord (**H,** arrowhead: pre-tangle-like inclusions), there were only a few fine granular tau deposits in the spinal anterior roots (**I** and **J**). (**K**) A small number of granular tau deposits is present in the IX/X nerve. (**A**) RD4, (**B**) RD3, (**C**) AT180, (**D**) pThr217, (**E**) Alz-50, (**F)** G-B, (**G**) p62 and (**H**–**K**) AT8. Scale bars in **A**–**C**(**i** and **ii**), **E**, **F**(**i** and **ii**), **G**(**i** and **ii**), **J** and **K** = 10 μm, **D** = 20 μm, **H** = 100 μm and **I** = 50 μm.

We subsequently evaluated the morphological features and staining profiles of tau pathology in the peripheral nerves of PSP and CBD cases (Figs 1 and 2). Additionally, the severity of tau lesions in the PNS was semi-quantitatively compared with the corresponding brainstem/spinal nuclei in PSP cases (Table 2). In PSP, p-tau deposits were observed in both intramedullary and extramedullary segments of cranial and spinal nerves (Fig. 1B, C and J). The p-tau-positive inclusions exhibited diffuse granular or elongated string-like structures (Fig. 1C and D). In cross-section, these inclusions appeared as granules or distinct inclusions located at the centre of the nerve fibres, namely the axons (Fig. 1F). The sagittal section revealed a mosaic pattern with granular regions and localized distinct inclusions, resembling beads (Fig. 1G). Additionally, occasional significantly large inclusions were present (Fig. 1H).

**Table 2:** Comparison of the severity of tau lesions in the PNS and the corresponding nuclei in PSP

Case ID	Cranial							Spinal					
	III n.	III nuc.	IX/X n.	X nuc.	Amb. nuc.	XII n.	XII nuc.	Anterior Root	AH	Region	Posterior Root	PH	Region
PSP1	++	++++	+++	++	+++	na	++++	++++	++++	C	+	++	C
PSP2	na	na	++	++	+++	++	+++	+	+++	C	na	++	C
PSP3	−	na	−	++	−	na	−	+	+	C	**+**	−	C
PSP4	+	na	na	++	++	na	++	+/+/**+**	++/+/−	C/T/L	−/−/−	++/+/+	C/T/L
PSP5	++	+++	+++	++	+++	na	++++	++++	++++	C	na	++	C
PSP6	na	na	++	++	+++	++++	+++	na	na		na	na	
PSP7	+	na	na	++	++	++	+++	na	na		na	na	
PSP8	na	+	na	na	na	na	na	**++**/+/**+**	−*/+/−	C/T/L	−/−/−	−*/−/+	C/T/L
PSP9	na	++	+	+++	+	+	++	++	++	C	++	++	C
PSP10	na	na	++	+	+++	na	++++	++++	+++	C	na	++	C
PSP11	na	na	++	++	++	++++	na	na	++++		na	++	C
PSP12	++	++	++	na	++	+++	na	+++	+++	C	na	++	C
PSP13	na	na	++	na	+++	na	na	+++	na	C	na	na	
PSP14	++	+++	na	na	++	na	na	na	na		na	na	
PSP15	+++	++++	+++	++	+++	na	++++	na	na		na	na	
Tau-positive/available number of cases (%)	7/8 (88%)	7/7 (100%)	10/11 (91%)	11/11 (100%)	13/14 (93%)	6/6 (100%)	9/10 (90%)	10/10 (100%)	10/10 (100%)		3/5 (60%)	9/10 (90%)	

For columns on the anterior and posterior roots we provide data on the cervical spinal cord levels; if different levels of the spinal cord were available, we indicate the results as follows: cervical level/thoracic level/lumbar level. The bold ‘+’ indicates that tau pathology was seen in the anterior or posterior root without tau pathology in the associated spinal cord area. n. = nerve; nuc. = nucleus; Amb. = ambiguous; AH = anterior horn; PH = posterior horn; C = cervical; T = thoracic; L = lumbar; na = not available. Semiquantitative assessment: − = absent; + = minimal; ++ = mild; +++ = moderate; ++++ = severe; −* = presence of only threads without neuronal cytoplasmic inclusions.

In contrast to PSP, CBD rarely exhibited tau deposits in the PNS despite showing severe tau pathology in the corresponding nuclei (Fig. 2H–K). Morphologically, PNS-tau lesions in CBD appeared as fine granular tau deposits (pre-tangle-like), which differed from the fibrillary large tau-positive inclusions (neurofibrillary tangle-like) observed in PSP. These morphological differences in PNS-tau pathology seem to reflect the differences in CNS-tau pathology between PSP and CBD. In addition, the 4-repeat tauopathy LNT case exhibited tau lesions in the PNS, and the severity was resembling PSP (Supplementary Fig. 1).

### Immunostaining profiles of the tau-positive inclusions in the PNS in progressive supranuclear palsy

The PNS-tau lesions, detected using the phosphorylated tau antibody (AT8), exhibited positivity for 4R-tau and negativity for 3R-tau (Fig. 2A and B and Supplementary Table 3). These findings were consistent with the CNS-tau pathology observed in PSP. The PNS-tau lesions also showed immunopositivity for several other phosphorylated tau antibodies (AT180, pThr217). Additionally, the lesions were recognized by Alz-50 (misfolded tau) antibody (Fig. 2C–E). The string-like and large structures with fibrillary morphology (neurofibrillary tangle-like) were argyrophilic with Gallyas silver staining. Some nerve fibres were filled with large p-tau immunoreactive inclusions, causing partial bulging (Fig. 2F). The p-tau-positive inclusions also showed immunopositivity for p62 (Fig. 2G and Supplementary Table 3).

### Double-labelling immunofluorescence analysis

**Figure 3:**
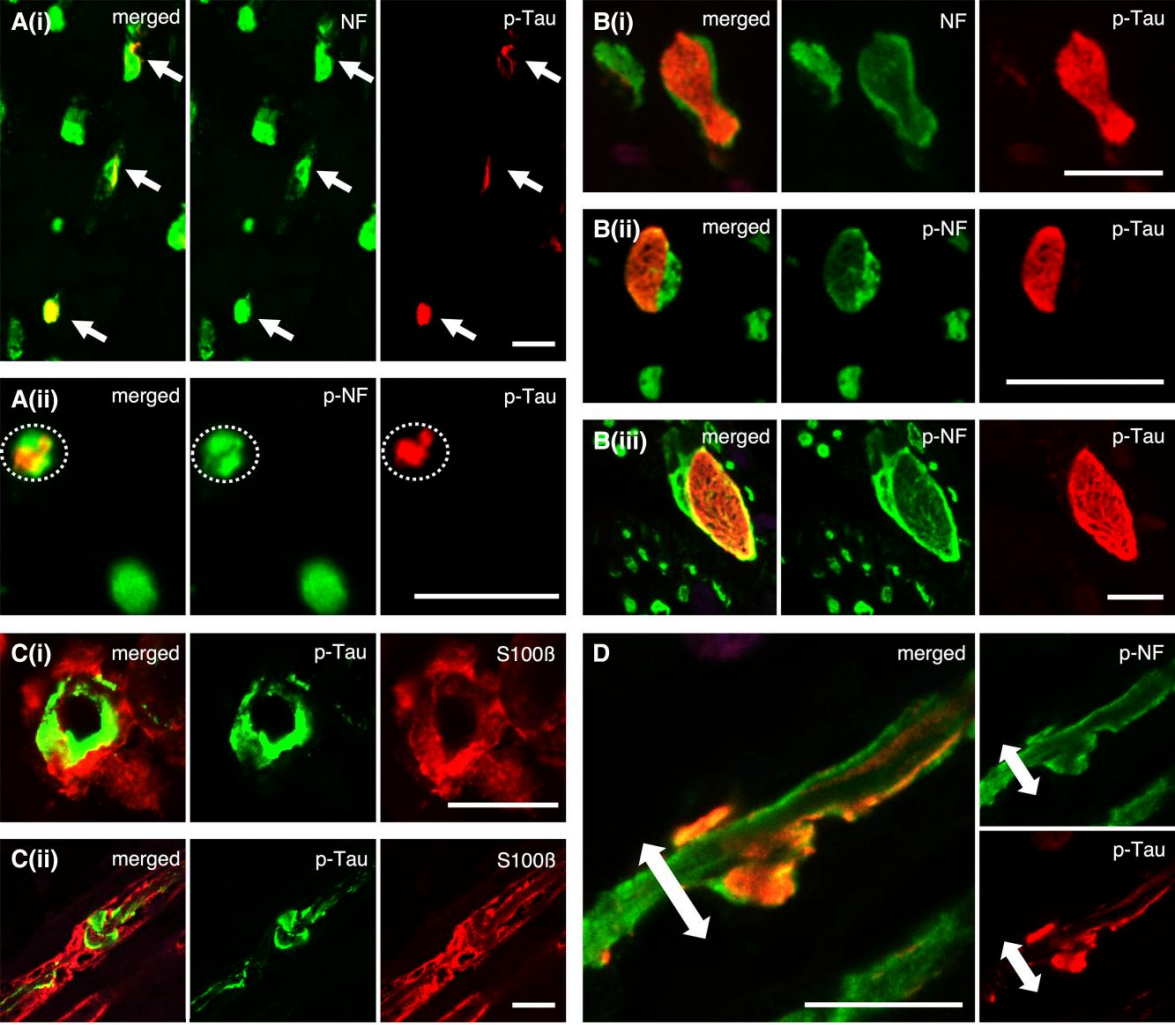
**Double-labelled immunofluorescence of phosphorylated-tau (p-tau) and neurofilament/S100β: the morphological features and localization sites of p-tau deposits in the peripheral nerve fibres**. (**A**) In cross sections, phosphorylated tau deposits are primarily localized within the neuronal fibres (**i**)/axons (**ii**). (**B**) Some of the nerve fibres are filled with large tau inclusions showing fibrillary morphology (**i–iii**). (**C**) A part of the large tau-positive inclusions appear to accumulate in the lateral portion of nerve fibres, suggesting the possibility that the deposits are partly located within myelin. However, p-tau and S100β are not completely co-localized, and p-tau deposits merely appear to overlap on the myelin [**C**(**i**): cross and **C**(**ii**): sagittal section]. (**D**) Sagittal section of D-IF: p-tau and p-NF. In support of this, p-tau deposits with the outer portion are present within the axons (p-NF). In addition, the axons detected with p-NF seem to be bulged due to the large tau-positive inclusions. [**A**(**i**) and **B**(**i**)] NF (green) and pThr217 (red), [**A**(**ii**), **B**(**ii** and **iii**) and **D**] SMI31 (green) and pThr217 (red) and (**C**) AT8 (green) and S100β (red). Scale bars in **A**–**D** = 10 μm.

Co-localization of p-tau immunoreactivity was assessed using double-labelling immunofluorescence analysis of p-tau and neurofilament protein (expressed in neuronal fibres and axons)/S100β (expressed in Schwann cells and myelin). This revealed that the p-tau-positive inclusions were present within the axons identified by NF and SMI31 (p-NF) antibodies (Fig. 3A).

This analysis also demonstrated the presence of large p-tau-positive inclusions with fibrillary morphology in the axons detected with NF and p-NF antibodies. These peripheral nerves were filled with large inclusions, resulting in partial expansion of the nerve fibre (Fig. 3B). This finding was consistent with the observations from immunohistochemistry and Gallyas silver staining [Figs 1H and 2A, E and F(ii)].

Immunohistochemical examination revealed some deposits primarily located externally, avoiding the centre of the nerve fibre [Figs 1K and 2G(i)] as if they were present in myelin/Schwann cells. However, in the double-labelling immunofluorescence, these p-tau deposits only partially overlapped with myelin/Schwann cells detected with S100β (Fig. 3C), and these lesions were observed just within the axons (Fig. 3D). Furthermore, the sagittal image also indicated that the peripheral nerve fibre was partially enlarged and displayed an altered morphological form due to the presence of tau inclusions (Fig. 3D). These results suggest that PNS-tau in PSP primarily accumulates within the axons, not in myelin/Schwann cells.

### Relationship between p-tau lesions in the nerves and corresponding brainstem nuclei and lower motor neurons

The cranial nerves III, IX/X, XII and spinal roots, which frequently exhibited PNS-tau lesions in PSP, were semi-quantitatively evaluated and compared to their corresponding nuclei, including the oculomotor nucleus, dorsal vagal nucleus, ambiguous nucleus, hypoglossal nucleus and spinal grey matter (anterior/posterior horn) in all PSP cases. In the spinal cord, the available cases were also evaluated at three levels: cervical, thoracic and lumbar cord. The severity of tau lesions in the PNS generally correlated with that in the corresponding nuclei (Table 2). For example, cases with severe tau lesions in the spinal anterior roots also showed severe tau involvement in the corresponding anterior horn (Fig. 1J and M).

Interestingly, when focusing on areas with minimal/mild tau pathology [e.g. PSP Case 4 (lumbar cord level) and PSP Case 8 (cervical/lumbar cord level)], there were no tau-positive inclusions in the neurons (sometimes, only a few tau-positive threads were present in the neuropil); however, tau deposits were clearly evident in the corresponding peripheral nerves (Table 2 and Supplementary Fig. 2).

### Extent of tau pathology in cranial and spinal nerves in progressive supranuclear palsy

The extent and severity of tau pathology in the evaluable cranial and spinal nerves were semi-quantitatively examined in all PSP cases (Table 3). The olfactory bulb (I) and cranial nerve II (CNS) were also included in the analysis. In the case of cranial nerves IV, VI and VII, where extramedullary nerve fibres were not available, tau pathology was evaluated within intramedullary nerve fibres.

**Table 3:** Distribution and severity of tau pathology in the PNS as well as co-pathology in 15 cases of progressive supranuclear palsy

	PSP Case	1	2	3	4	5	6	7	8	9	10	11	12	13	14	15	Tau (+)/available number of cases (%)
Cranial	I (Olf. bulb)	na	na	na	−	na	++	na	na	na	na	++	++	na	na	na	3/4 (75)
	II	na	na	na	na	+	+	na	na	na	na	+	na	na	na	na	3/3 (100)
	III	++	na	−	+	++	na	+	na	na	na	na	++	na	++	+++	7/8 (88)
	IV, intra^a^	na	na	na	na	na	na	na	na	na	na	++++	na	++	na	na	2/2 (100)
	V	na	na	na	na	na	na	na	na	na	+++	na	na	na	na	na	1/1 (100)
	VI, intra^a^	na	na	na	na	na	+++	na	++	na	na	na	na	na	na	na	2/2 (100)
	VII, intra^a^	na	na	na	na	na	+++	na	++	na	na	na	na	na	na	na	2/2 (100)
	IX/X	+++	++	−	na	+++	++	na	na	+	++	++	++	++	na	+++	10/11 (91)
	XI	na	na	na	na	na	na	++	na	na	+++	+++	+++	na	na	na	4/4 (100)
	XII	na	++	na	na	na	++++	++	na	+	na	++++	+++	na	na	na	6/6 (100)
Spinal (A/P)	Cervical	++++/+	+/na	+/+	+/−	++++/na	na	na	++/−	++/++	++++/na	na	+++/na	+++/na	na	na	A:10/10(100), P: 3/5 (60)
	Thoracic	na	na	na	+/−	na	na	na	+/−	na	na	na	na	na	na	na	A: 2/2 (100), P: 0/2 (0)
	Lumbar	na	na	na	+/−	na	na	na	+/−	na	na	na	na	na	na	na	A: 2/2 (100), P: 0/2 (0)
	Cauda equina	na	na	na	−	na	na	na	−	na	na	na	na	na	na	na	0/2 (0)
Tau-positive/available number of areas (%)		3/3 (100)	3/3 (100)	1/3 (33)	4/6 (66)	4/4 (100)	6/6 (100)	3/3 (100)	5/6 (83)	3/3 (100)	4/4 (100)	6/6 (100)	6/6 (100)	3/3 (100)	1/1 (100)	2/2 (100)	
**Other features**																	**TDP-43 (+)/available number of cases (%)**
pTDP-43 (CNS)	Motor area	−	−	−	+	−	−	−	−	−	−	−	−	−	−	−	1/15 (6.7)
	Spinal cord	−	na	−	+	−	na	na	+	−	−	na	−	na	na	na	2/8 (25)
Co-pathology	AD (15)	Not	Not	Int.	Not	Not	Not	Not	Int.	Low	Not	Not	Low	Low	Not	Low	
	AGD (18)	0	0	0	3	0	3	0	3	1	0	1	1	0	0	0	
	LATE (20)	0	0	0	0	0	0	0	1	0	0	0	0	0	0	0	
	LBD (19)	0	1	4	0	0	0	0	0	0	2	0	0	1	0	0	

A = anterior spinal roots; AD = Alzheimer’s disease; AGD = argyrophilic grain disease; Int. = intermediate; intra = intramedullary; LATE = limbic predominant age-related TDP-43 encephalopathy; LBD = Lewy body disease; na = not available; Olf = olfactory; P = posterior spinal roots. Semiquantitative assessment: − = absent; + = minimal; ++ = mild; +++ = moderate; ++++ = severe.

^a^A part of cranial nerves (IV, VI and VII) was only evaluated in the intramedullary nerve fibres.

The results showed that cranial nerves I (olfactory bulb) and II (CNS), as well as IV, VI and VII (intramedullary), often exhibited p-tau deposits, with the following observations: I (3/4: 75%), II (3/3: 100%), IV (2/2: 100%), VI (2/2: 100%) and VII (2/2: 100%). The accumulation of tau in cranial nerve II was minimal but present. In the olfactory bulb, tau pathology was also present in PSP cases where AD neuropathological changes were ‘Not’ or ‘Low’ (Supplementary Fig. 3). Upon analysing each case individually, tau lesions frequently affected multiple cranial and spinal nerves. The degree of tau pathology tended to be particularly severe in the anterior spinal roots and cranial nerve XII, followed by cranial nerves IX/X and XI, namely at the levels of lower brainstem and spinal cord.

### Tau immuno-blot analysis and *in vitro* tau seeding assay

**Figure 4:**
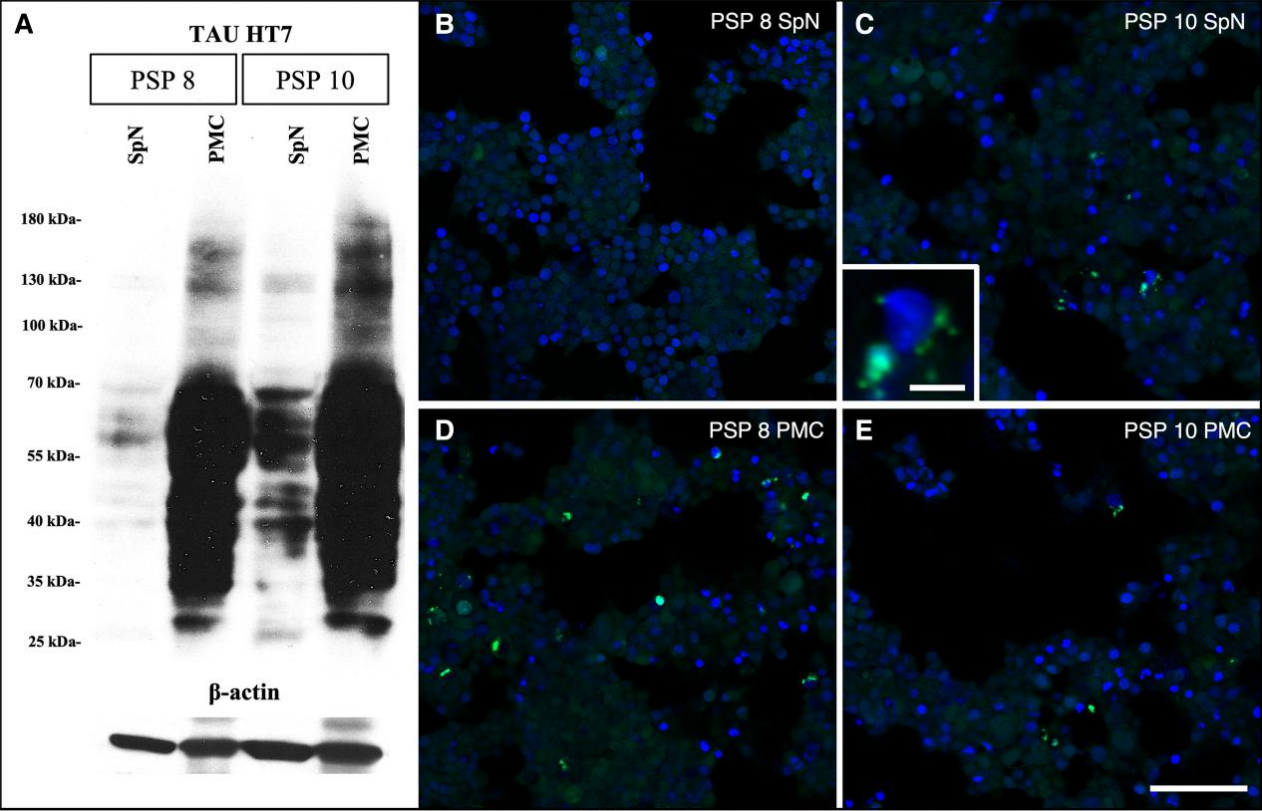
**Tau immunoblot analysis and *in vitro* tau seeding assay.** (**A**) Tau immunoblot, total tau (HT7 clone), reveals the presence of tau in all four samples. (**B**–**E**) *In vitro* tau biosensor cells demonstrate that tau deposits (green) in the primary motor cortex (**D** and **E**) and cervical spinal nerve (**C**, enlarged image in the *bottom left* corner) samples have seeding capacity. (**B** and **D**) PSP Case 8 (PSP 8) and (**C** and **E**) PSP Case 10 (PSP 10). PMC = primary motor cortex; SpN = spinal nerves. Scale bars in **B**–**E** = 100 μm and **C**, enlarged image in the *bottom left* corner = 10 μm.

Frozen samples of cervical spinal roots were available for two PSP cases (PSP Cases 8 and 10). Frozen samples were also taken from the motor cortex. Western blotting revealed the expression of tau in these four samples (two cases, two areas) (Fig. 4A). Subsequently, an *in vitro* tau seeding assay was conducted. In PSP Case 10, which exhibited more abundant tau deposits in the cervical anterior spinal roots, p-tau deposits present in the PNS had seeding capacity, in addition to that in the CNS (Fig. 4B–E).

### TDP-43 pathology in progressive supranuclear palsy

Given the recent reports of TDP-43 pathology in PSP, particularly in motor areas such as the motor cortex and spinal cord^30^ and our observation on tau lesions in the motor nerves in PSP cases, we also assessed the presence of TDP-43 pathology in all PSP cases, with a focus on motor areas. As a result, a few p-TDP-43-positive inclusions were observed in 2 of 15 PSP cases (13.3%), specifically in the motor cortex (1/15, 6.7%) and spinal cord (2/8, 25%). Phospho-TDP-43-immunoreactive inclusions were identified as neuronal inclusions and threads in the motor cortex, while threads and glial inclusions were observed in the spinal white matter (Table 3 and Supplementary Fig. 4). Phospho-TDP-43-immunoreactive inclusions were not found in the anterior horn cells, spinal roots or cranial nerves (Supplementary Table 4). Among the two p-TDP-43-positive cases, one case additionally showed limbic-predominant age-related TDP-43 encephalopathy (LATE) stage 1.^20^ These findings suggest that TDP-43 pathology is not strongly associated with PNS-tau pathology in PSP (Table 3).

### Clinical presentation of all studied tauopathies: peripheral neurological symptoms

Table 4 summarizes the clinical presentation of all studied tauopathies (PSP, AD, CTE and CBD), with special reference to peripheral neurological symptoms. We particularly examined eye movement disorders, bulbar symptoms and motor/sensory palsy in the extremities, considering that the cranial nerves III, IX/X, XII and spinal roots showed high tau accumulation. The results revealed PSP cases presented with a high incidence of bulbar symptoms and eye movement disorder, unlike other tauopathies. Interestingly, some PSP cases with PNS-tau lesions in the anterior spinal roots were also accompanied by lower-motor neuron signs (Tables 3 and 4 and Supplementary Table 5).

**Table 4:** Clinical summary of the progressive supranuclear palsy, Alzheimer’s disease, chronic traumatic encephalopathy and corticobasal degeneration cases studied

	PSP (*n* = 15)	AD (*n* = 18)	CTE (*n* = 5)	CBD (*n* = 6)
Sex, male: female	10:5	9:9	5:0	4:2
Age at death, average ± SD	74.7 ± 7.8	70.1 ± 10.0	70.4 ± 18.5	70.0 ± 9.6
Duration of illness, years, average ± SD	8.0 ± 5.0	8.3 ± 3.7	10.7 ± 12.0	9.5 ± 5.9
**Symptoms [positive/available number of cases (%)]**				
Eye movement disorder	**13/15 (87)**	3/13 (23)*	2/5 (40)	2/4 (50)
Supranuclear palsy (oculocephalic manoeuvre^a^)	11/11^a^	3/3	2/2	0/1*
Bulbar palsy	**14/14 (100)**	2/13 (15)***	0/5 (0)**	2/4 (50)
Sensory disturbance in the extremities	5/15 (33)	3/11 (27)	1/5 (20)	1/3 (33)
Motor paralysis in the extremities	5/14 (36)	5/13 (38)	2/5 (40)	0/2 (0)

Pick’s disease cases (age range 54–75 years) are not included since clinical information on eye movement disorder or bulbar palsy was not available. The bold indicates symptoms with a high incidence in PSP cases, compared to other tauopathies. AD = Alzheimer’s disease; CBD = corticobasal degeneration; CTE = chronic traumatic encephalopathy; PSP = progressive supranuclear palsy; SD = standard deviation. *P < 0.05; **P < 0.01; ***P < 0.0001 (versus PSP cases, Fisher’s exact test).

^a^Documented well before death; the patients’ supranuclear palsy may have evolved to a nuclear palsy late in the course,^31^ but this was not documented in the records.

## Discussion

This study provides the first evidence of distinct p-tau immunoreactive structures in the peripheral nerves of tauopathies, with significantly greater involvement in PSP compared to other tauopathies (Table 1). All studied PSP cases exhibited tau accumulation in cranial or spinal nerves, particularly in cranial nerves III, IX/X, XII, and anterior roots. Additionally, the tau biosensor assay demonstrated, for the first time, that pathological tau not only in the CNS but also in the peripheral nerves possess seeding capacity.

While our understanding of tau aggregation in peripheral nerve tissues is still limited in histopathological studies,^11-13^ it has been well documented in α-synucleinopathies.^5^ In PD/LBD, α-synuclein predominantly accumulates in the axons,^32^ and in multiple system atrophy (MSA), it accumulates in Schwann cells.^21^ Furthermore, animal studies have suggested that α-synuclein may accumulate in the PNS before affecting the corresponding nuclei in the CNS.^33,34^ In addition, these models have been shown to result in associated peripheral neurological symptoms.^35,36^

PNS-tau in PSP exhibited 4R-tau with an immunoreactivity profile for different anti-tau antibodies resembling the neurofibrillary tangles observed in the CNS. PNS-tau in CBD displayed morphological features with fine diffuse/granular structures, resembling the p-tau deposition pattern seen in neurons.^1,37^ Thus, tau lesions in the PNS reflect well with the morphology of tau pathology in the CNS. On the other hand, the frequency of PNS-tau was significantly different between PSP and CBD.

The underlying mechanisms for the differences in why PNS-tau is observed only in 4R-tauopathies such as PSP and CBD but not AD, CTE or Pick’s disease and why PNS-tau is more prevalent and prominent in PSP are unclear. Analogously, the distinct neuronal vulnerability patterns among tauopathies have also not yet been completely elucidated. However, a recent study suggests that tauopathies can be distinguished based on the structure of tau filaments.^3^ We can speculate that, similarly to CNS-tau, differences in the conformational properties of misfolded tau and predominance of tau isoforms, as seen also in PSP and CBD,^3^ may be related to the susceptibility of tau lesions to peripheral nerves, namely, the pathological tau in PSP may be more prone to invade PNS. Indeed, we additionally evaluated some cases of Pick’s disease (3R-tauopathy) and one further case with the 4-repeat tauopathy, LNT.^14^ Interestingly, we found no PNS-tau lesions in the Pick’s disease cases (Supplementary Table 2), while p-tau-positive deposits were present in the PNS in LNT (Supplementary Fig. 1). In the latter case, the severity of tau pathology in the PNS resembled PSP rather than CBD cases. Interestingly, it has been reported that LNT is hierarchically closer to PSP than CBD in terms of tau filaments^3^ and CNS histopathology.^14^ Furthermore, it may also be necessary to investigate the relationship between PNS-tau and one of the special tau isoforms that are abundantly expressed in the PNS, the so-called ‘big tau’.^38-40^ Interestingly, big tau has been reported as 4R-tau.^41,42^

This study shows that tau accumulation is predominantly in axons and not in the myelin or Schwann cells. This result differs from α-synuclein; Schwann cells are involved in MSA,^21^ while in LBD the accumulation in the PNS occurs predominantly in axons with minimal involvement of the Schwann cells.^32^ Therefore, neurodegenerative proteins show distinct selective vulnerability of different components of the PNS.

Importantly, in PSP, the severity of p-tau lesions in the cranial and spinal nerves generally correlated with that in the corresponding nuclei; however, focal p-tau deposits were present in some nerves without involvement in the corresponding nuclei. On the other hand, in CBD, we observed tau pathology in lower motor neurons without or only with mild involvement of anterior spinal roots. These observations suggest that (i) propagation of disease-associated tau into the periphery depends on the type of fibrillar tau; or (ii) that p-tau lesions in peripheral nerves are not necessarily secondary to the corresponding nuclei. We cannot exclude the possibility that tau accumulation starts in the periphery in some cases, although the lack of fibrillar tau pathology in peripheral organs does not support this concept.^12,13^

Interestingly, PNS-tau was observed predominantly in peripheral motor nerves in PSP. A recent study reported that PSP cases frequently show TDP-43 pathology in lower (38%) and upper (19%) motor neurons.^30^ Although in our collection we could not find a similar frequency (upper motor neuron: 6.7%; lower motor neuron: there were only a few neurites and positive glia), together with further studies describing involvement of lower motor neurons in PSP,^11^ these observations emphasize the vulnerability of the motor system in 4R tauopathies. Indeed, another 4R tauopathy, globular glial tauopathy,^43,44^ is a disorder where this is more prominent. Importantly, globular glial tauopathies show overlap with PSP, exemplified by the type II subtype, and also the structure of tau filaments shows similar features.^3^ When comparing PSP cases with and without TDP-43 pathology, no remarkable differences in the extent or degree of PNS-tau pathology were found. One of the two cases also showed LATE-neuropathologic change. However, the previous study examining TDP-43 pathology in PSP documented that the presence or absence of spinal cord TDP-43 pathology was not significantly associated with LATE stage and that extra-limbic TDP-43 pathology differs from the spreading manner of LATE.^20,30^ Altogether we concluded that no association between PNS-tau and TDP-43 pathology can be confirmed in our study.

We also examined the extent of tau pathology in the PNS within each PSP case. Although cranial nerves I (olfactory bulb) and II (optic chiasm and nerve) are part of the CNS, they were included in the analysis. Strikingly, all PSP cases showed extensive tau accumulation in multiple cranial nerves, including the olfactory bulb and optic nerve, and spinal anterior/posterior roots from cervical to lumbar cord levels. Notably, even cases with relatively short disease durations (PSP Cases 2 and 6, with 3 years duration of illness), exhibited p-tau lesions in multiple nerves supporting the notion that PNS-tau lesions may occur relatively early in the disease progression of PSP. Tau accumulation in the olfactory bulb is often associated with tauopathy, including PSP and particularly AD.^45^ Although the number of cases available for evaluation was small, we assessed tau accumulation in the optic nerve and found it to be present in all examined PSP cases (3/3). This is compatible with findings of the involvement of the visual system in AD and PSP^46^ and with a recent study that reported tau accumulation in the retina as a diagnostic biomarker for tauopathies.^47^

Although, motor nerves were predominantly affected, involvement of the IX/Xth (91%) and Vth (100%) cranial nerve and the spinal posterior roots (60%) indicate that the sensory nervous system is also involved in the disease process of PSP. These observations are important for the potential of skin biopsy as a biomarker also for tauopathies. Importantly, we show that tau in the spinal roots has seeding ability. Previous studies have predominantly focused on the involvement of the PNS in α-synucleinopathies. PD frequently presents with non-motor symptoms, including sensory and autonomic disturbances, and pathologically the aggregation of pathological proteins in peripheral nerves is a usual feature in α-synucleinopathies.^4,5^ Recently, RT-QuIC seeding activity of α-synuclein in the skin has been shown to have potential diagnostic value in synucleinopathies.^6,7^

Despite the paucity of observations on tau pathology in nerves, recent studies have begun to capture involvement of the PNS in tauopathies. Indeed, autonomic and sensory disturbances, with electrophysiological and pathological features have been reported in PSP.^8-10^ Histopathologically, PSP patients displayed a length-dependent loss of sensory and autonomic nerve fibres in the skin biopsy samples.^9^ Interestingly, the P301L mutant tau transgenic animal model exhibits motor deficits with severe peripheral nerve involvement, such as axonal spheroids in the anterior spinal roots, axonal degeneration in peripheral nerves and neurogenic skeletal muscle atrophy,^48^ supporting the notion of peripheral involvement in tau-related diseases. However, the findings related to the expression of p-tau immunoreactivity in peripheral nerves are still limited in the previous studies.^5,11-13^ In the spinal ganglia, no NFTs were detected in eight patients with PSP and 20 patients with AD.^5^ Stejskalova *et al*.^11^ reported p-tau-immunoreactivity (AT8) in the anterior and posterior roots in the PSP cases. Dugger *et al*.^12^ examined abdominal skin, submandibular gland and sigmoid colon in the PSP cases. Phospho-tau-immunoreactivity (AT8) of those nerve elements was very rare. Recently, in the peripheral nerves of skin, increased tau mRNA expression and total tau protein in the PSP cases has been reported,^13^ suggesting its potential as a biomarker.^6,7^ However, even in that study, immunostaining (immunofluorescence) did not demonstrate positivity for phosphorylated tau.^13^ This may have been due to a small number of peripheral nerve tissues and a few tau depositions in the skin. Our immunohistological analysis did not reveal clear tau aggregation in peripheral nerves in cases of AD and CTE, while there is a report of tau expression in peripheral tissues in AD.^12^ The possibility that peripheral tissues in tauopathies other than PSP could also be used as biomarkers for diagnostic purposes merits further studies.

Regarding clinical features, not surprisingly we observed a significantly higher frequency of eye movement disorders and bulbar symptoms in PSP cases compared with other tauopathies. Interestingly, some PSP cases with PNS-tau lesions in the anterior spinal roots were also accompanied by lower-motor neuron signs. However, cranial nerve palsy is not a classical feature of PSP and was not detected in our cohort. Therefore, based on the limited retrospective clinical data in this study, we cannot conclude that these symptoms were truly due to peripheral nerve impairment. While prospective studies can identify PNS-related symptoms, the message of finding PNS-tau pathology is more to support the notion that detection of misfolded tau in the PNS can be a target for biomarker development, such as implemented for the α-synucleinopathies, for example using skin biopsies.

This study has several limitations. First, peripheral neurological symptoms were evaluated retrospectively. A larger comprehensive and prospective continuous assessment of peripheral neuropathy may be necessary to accurately evaluate its presence in tauopathies. Second, the samples we used did not allow assessment of a mild reduction and degeneration of peripheral nerve fibres. Prominent loss of nerve fibres, such as in motor neuron disease or hereditary neuropathies, was not seen in the PSP cases examined. However, the presence of previously reported peripheral nerve loss and symptoms, abnormalities in nerve conduction velocity,^9,10^ increased expression of tau protein in the PNS,^13^ and the presence of large tau inclusions and p-tau deposits filling many nerve fibres in this study suggest that accumulated tau in the peripheral nerve fibres is likely to cause some dysfunction of the peripheral nerves. Third, skin and peripheral ganglia were not evaluated. Evaluation of skin is necessary for future biomarker studies. Since the amount of pathological tau might be too low to be consistently detected by immunohistochemistry, seeding amplification assays (RT-QuIC) might be a more feasible approach.

In conclusion, in this study, we showed for the first time that tau pathology with distinct p-tau immunoreactive fibrillar structures is evident in the cranial nerves and spinal nerve roots in PSP and to a far lesser extent in CBD. The morphological characteristics of PNS-tau resemble those in the CNS and show selectivity for PSP. Furthermore, we showed for the first time that tau in peripheral nerves has seeding ability using tau biosensor cells. Tau pathology in PNS is a previously unappreciated feature of tauopathies that could serve as a potential diagnostic biomarker in the future.

## Supplementary Material

awad381_Supplementary_Data

## Data Availability

The authors confirm that the data supporting the findings of this study are available within the article and its Supplementary material.
