# Supplementary material for: Distinct involvement of the cranial and spinal nerves in progressive supranuclear palsy
Source: Brain. 2023 Nov 16;147(4):1399–411. doi: 10.1093/brain/awad381 (PMC10994524; doi:10.1093/brain/awad381)
Supplement: awad381_Supplementary_Data [file awad381_supplementary_data.pdf]

Supplementary Materials for:

Distinct involvement of the cranial and spinal nerves in progressive supranuclear palsy

Hidetomo Tanaka, Ivan Martinez-Valbuena, Shelley L. Forrest, Blas Couto, Nikolai Gil Reyes, Alonso Morales-Rivero, Seojin Lee, Jun Li, Ali M. Karakani, David F. Tang-Wai, Charles Tator, Mozhgan Khadadadi, Nusrat Sadia, Maria Carmela Tartaglia, Anthony E. Lang, and Gabor G. Kovacs

The supplementary materials contain five s-Tables and four s-Figures.

Supplementary Table 1. Summary of the co-pathology of the studied cases

	PSP (n = 15)	AD (n = 18)	CTE (n = 5)	CBD (n = 6)
AD-NC score*, mean (± SD)	0.53 (±0.74)	3.00 (±0.00)	0.80 (±1.30)	0.80 (±0.84)
AGD positive, n (%)	6 (40.0)	0 (0.0)	3 (60.0)	2 (33.3)
LBD positive, n (%)	4 (26.7)	13 (72.2)	2 (40.0)	2 (33.3)
LATE-NC positive, n (%)	1 (6.7)	5 (27.8)	2 (40.0)	1 (16.7)

AGD = argyrophilic grain disease; LBD = Lewy body disease; LATE-NC = limbic predominant age-related TDP-43 encephalopathy neuropathological change; SD = standard deviation. *AD-NC score: the level of AD neuropathologic change[15] was scored as follows, Not = 0; Low = 1; Intermediate = 2; High = 3.

Supplementary Table 2. Summary of tau pathology in the PNS in Pick's disease

		Pick's disease (n = 9)		
**# of cases**		**Available**	**Tau**	**%**
**Cranial nerves**				
	**III**	3	0	**0**
	V	1	0	**0**
	**IX/X**	2	0	**0**
	**XII**	4	0	**0**
**Spinal roots**				
	**Ant.**	1	0	**0**
	Post.	1	0	**0**
**Total case #**		5	0	**0**

Available = number of available cases; Tau = number of tau-positive cases; Ant. = anterior spinal roots; Post. = posterior spinal roots.

Supplementary Table 3. Summary of staining profiles of the PNS-tau in PSP cases

Staining profile of PNS-tau							
**4R-tau**	**3R-tau**	**AT8**	**AT180**	**pThr217**	**Alz-50**	**Gallyas**	**p62**
++	-	++	++	++	+	+ ~ ++	+

They are evaluated using the following criteria. -, negative; +, positive; ++, positive (abundant).

Supplementary Table 4. Summary of p-TDP-43 pathology in the PNS in PSP

	PSP (n = 15)		
# of cases	Available	TDP-43	%
**Cranial nerves**			
**III**	3	0	**0**
V	1	0	**0**
**IX/X**	2	0	**0**
**XII**	4	0	**0**
**Spinal roots**			
**Ant.**	1	0	**0**
Post.	1	0	**0**
**Total case #**	5	0	**0**

PSP = progressive supranuclear palsy; Available = number of available cases; TDP-43 = number of p-TDP-43-positive cases; Ant. = anterior spinal roots; Post. = posterior spinal roots.

Supplementary Table 5. Clinical features of all studied PSP cases

Case No.	1	2	3	4	5	6	7	8	9	10	11	12	13	14	15	
Sex	F	F	F	M	M	M	M	M	M	M	M	F	M	F	M	M:F = 10 : 5
Age at death	69	63	90	73	76	73	71	93	77	72	72	73	79	68	72	74.7 ± 7.8
Duration of illness, years	8	3	23	8	5	3	5	8	8	11	11	7	5	4	11	8.0 ± 5.0
Clinical diagnosis	PSP	PSP-F	PDD	CTE	PSP-RS	PSP-RS	PSP-RS	CBS	PSP-RS	PSP-RS	PSP	CBS	PSP-RS	PSP	PSP	
Symptoms																# of positive/available cases (%)
Eye movement disorder	+	+	-	+	+	+	+	-	+	+	+	+	+	+	+	13/15 (87%)
- Supranuclear palsy (Oculocephalic maneuver*)	+	+	na	+	+	+	na	na	+	na	+	+	+	+	+	11/11 (100)
Bulbar palsy	+	+	+	na	+	+	+	+	+	+	+	+	+	+	+	14/14 (100)
-Dysphagia	+	+	+	na	+	+	+	+	+	+	+	-	+	-	+	12/14 (86)
-Dysarthria	+	na	+	na	+	+	+	na	+	+	+	+	+	+	+	12/12 (100)
Sensory disturbance in the extremities	-	-	+	+	-	+	-	-	-	-	+	-	-	-	+	5/15 (33)
Motor paralysis in the extremities	-	+	-	+	-	-	na	+	-	-	-	+	-	+	-	5/14 (36)
Lower-motor neuron signs	-	na	na	+	+	+	na	na	+	-	+	na	-	na	+	6/9 (67)
Other features	Urinary urgency				Urinary frequency; diplopia, eyelid dysfunction	Diplopia					Urge incontinence, diplopia, DM					

M = male; F = female; CBS = corticobasal syndrome; PDD = Parkinson's disease with dementia; PSP-RS = PSP-Richardson's syndrome; PSP-F = PSP-frontal variant; na = not available.

*Documented well before death; the patients' supranuclear palsy may have evolved to a nuclear palsy late in the course,[31] but this was not documented in the records.

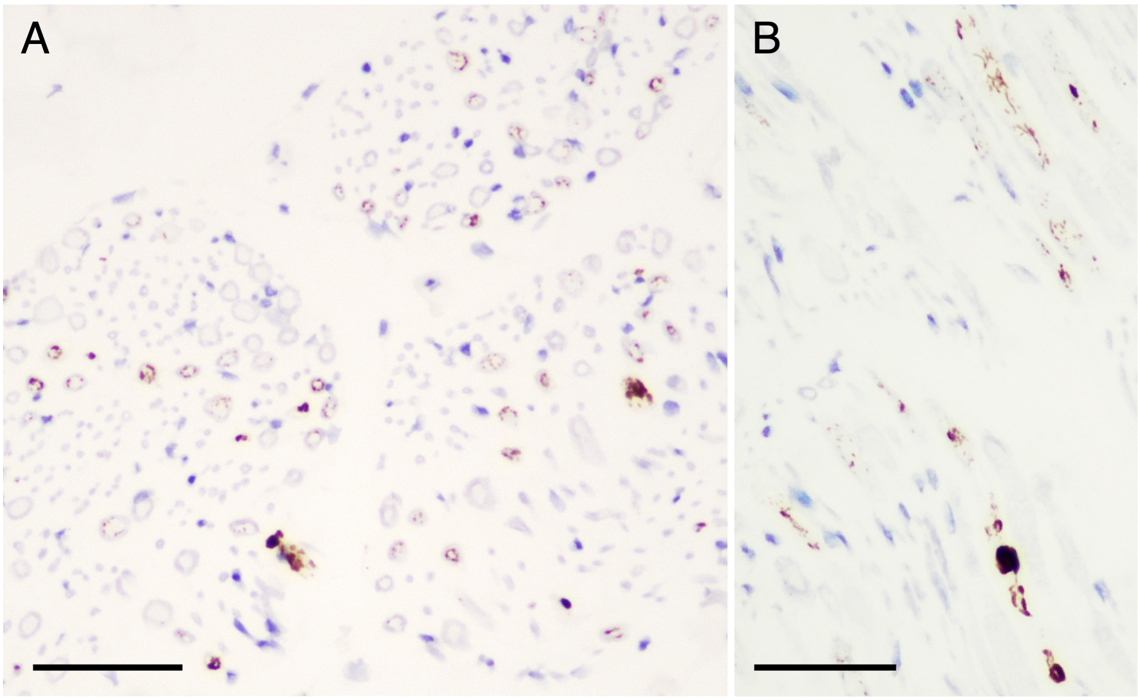

Supplementary Figure 1. PNS tau lesions in the further case with limbic-predominant neuronal inclusion body 4-repeat tauopathy (LNT)/PSP-type pathology.

(**A, B**) p-tau pathology (AT8) in the spinal anterior roots, cross sections (**A**) and sagittal sections (**B**). Scale bars: (**A** and **B**) 50 μm.

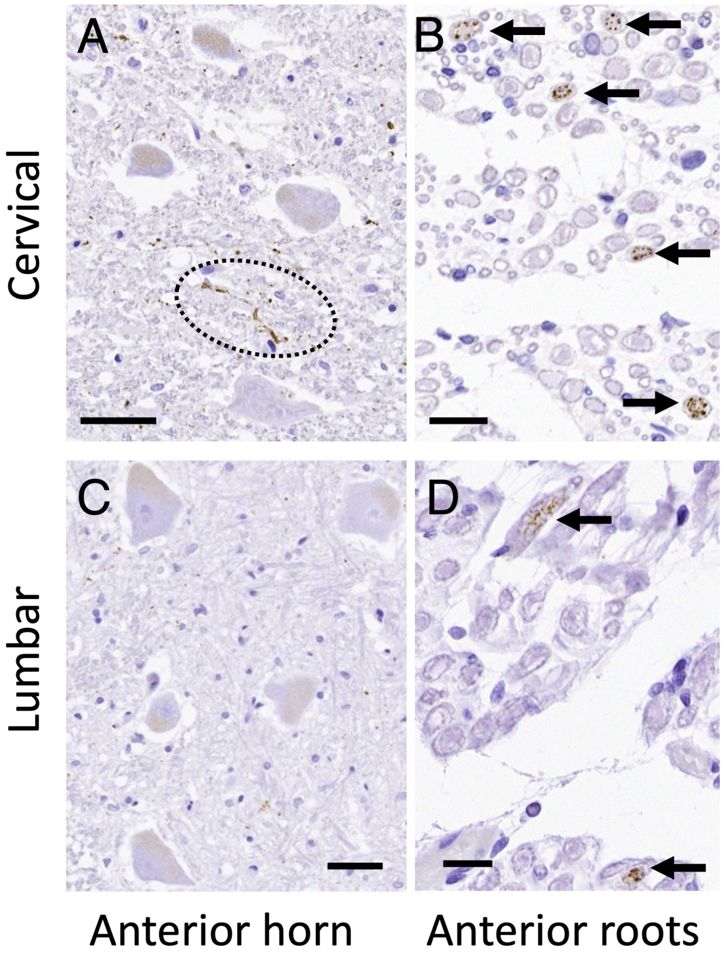

Supplementary Figure 2. Tau accumulation in the anterior spinal root without neuronal cytoplasmic inclusions in the corresponding anterior horn.

(**A-D**) While there are only few threads (**A**: cervical cord, circle) and no obvious tau-positive inclusions (**C**: lumbar cord), tau aggregates are shown in the anterior spinal roots (**B**: cervical and **D**: lumbar; arrows: tau aggregates). (**A-D**) PSP case 8. (**A-D**) AT8. Scale bars: (**A** and **C**) 50 μm, (**B** and **D**) 20 μm.

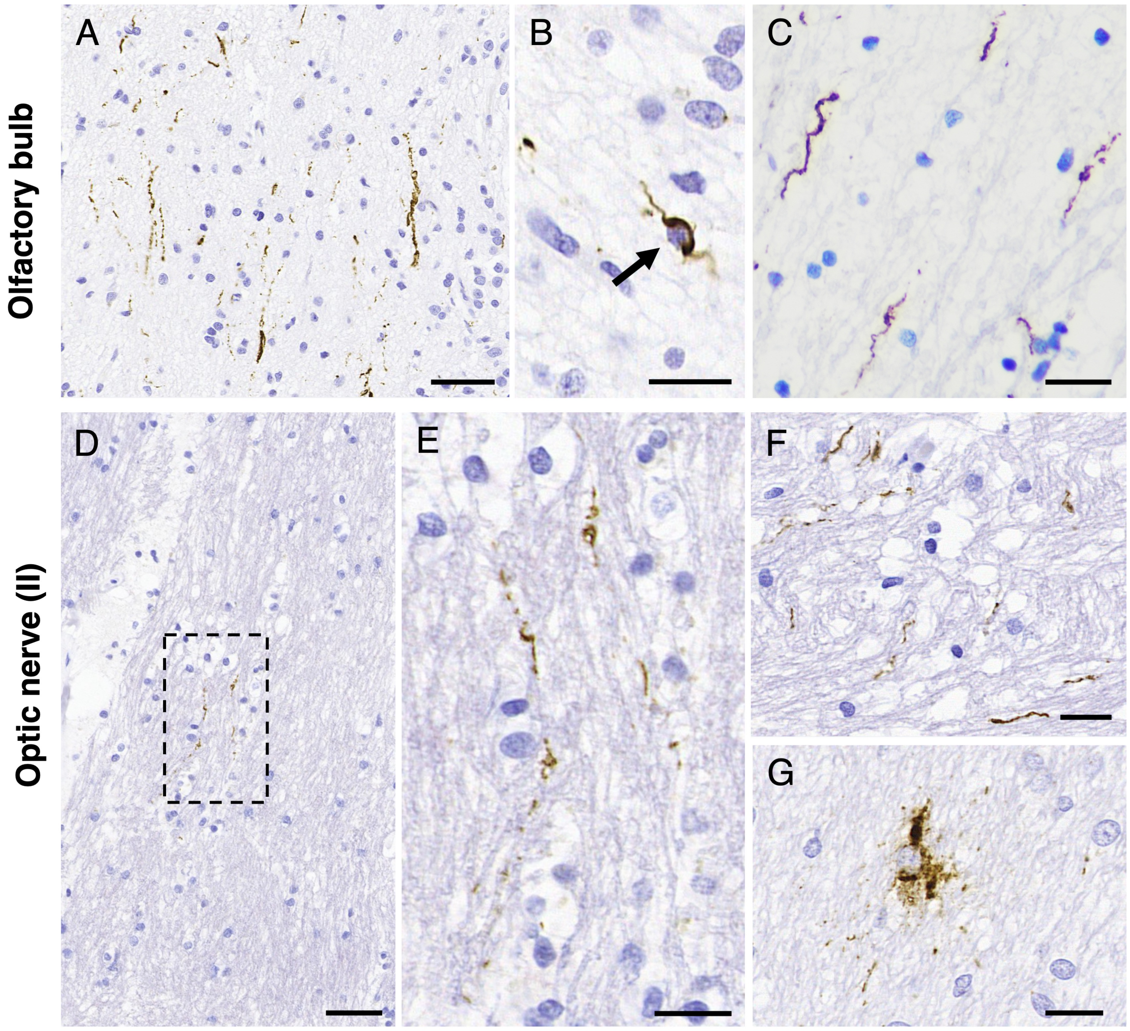

Supplementary Figure 3. p-tau (AT8)-positive inclusions in the olfactory bulb and optic nerves in PSP cases.

(**A-C**) p-tau-positive inclusions in the olfactory bulb, including threads, neurites (**A** and **C**), and oligodendroglial coiled body-like inclusions (**B, arrow**). (**D-G**) p-tau-positive inclusions in the optic nerve (**D**), including threads, neurites (**E:** high magnification image of clear square in **D;** and **F**), and tau-positive cytoplasmic inclusions resembling tau-positive astrocytes (**G**). (**A, B**, and **G**) PSP case 6, (**C** and **F**) PSP case 11, (**D** and **E**) PSP case 5. Scale bars: (**A** and **D**) 50 μm, (**B, C, E, F,** and **G**) 20 μm.

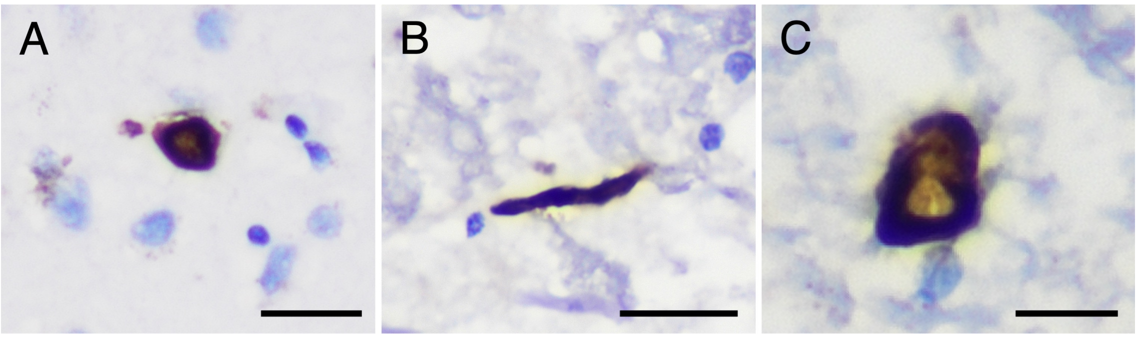

Supplementary Figure 4. pTDP-43-positive inclusions in the CNS, motor cortex and spinal cord, in the PSP cases.

(**A-C**) pTDP-43-positive inclusion in a neuron (**A**), neurite (**B**), and glial cell (**C**) in the motor cortex (**A**) and spinal cord white matter (**B** and **C**). Scale bars: (**A** and **B**) 20 μm, (**C**) 10 μm.
